# Comparison of Efficacy Between Vaginal Sildenafil and Granulocyte-Colony Stimulating Factor (G-CSF) in Improving Endometrial Thickness (ET) in Infertile Women

**DOI:** 10.7759/cureus.26415

**Published:** 2022-06-29

**Authors:** Parth Belapurkar, Arpita Jaiswal, Sparsh Madaan

**Affiliations:** 1 Department of Obstetrics and Gynecology, Datta Meghe Institute of Medical Science, Jawaharlal Nehru Medical College, Wardha, IND

**Keywords:** efficacy, endometrial thickness (et), granulocyte-colony stimulating factor, sildenafil, infertility

## Abstract

Background

Infertility is presently an emanating preventive medicine issue with some severe societal repercussions associated with it. In India, approximately a score percent of couples bear the burden of infertility. Moreover, the declining fertility rates despite effective artificial reproductive techniques and increasing development of modern reproductive medicine from the last two censuses pose an alarm to the demographic progression data. Many studies have highlighted the importance of shifting the research focus to endometrial receptivity for increasing clinical pregnancy.

Objective

This research aims to compare the efficacy of treatments of vaginal sildenafil citrate and granulocyte-colony stimulating factor (G-CSF) intrauterine injection in increasing endometrial thickness (ET).

Methodology

This was a randomized control trial (RCT) conducted over a two months period. Women seeking infertility treatment were recruited from the hospital's gynecological outpatient department (OPD). After the subjects gave informed consent, their history, clinical examination, and investigations were assessed. From the sixth day of the menstrual cycle, group A and group B had serial trans-vaginal ultra-sonographic evaluations for baseline endometrial thickness measurements. From day six to day 12 of the menstrual cycle, patients of group A were requested to self-administer per vaginal sildenafil citrate 25 mg every six hours. ET was evaluated sonographically on day 12 and day 14 of their menstrual cycle. Patients of group B received G-CSF 300 mcg/ml *as *intrauterine instillation on day 10 and were evaluated sonographically on day 12 and day 14 of their menstrual cycle. Patients then underwent additional therapy in the form of intrauterine injection (IUI), intracytoplasmic sperm injection with/without embryo transfer (ICSI/ET), or a natural cycle. Paired as well as unpaired t-tests were applied to the study groups to detect significant differences in the measurement of endometrial thickness before and after treatment.

Results

It was noticed that both sildenafil and G-CSF are agents for increasing endometrial thickness. The mean increase in endometrial thickness in the sildenafil treated group was 3.87 mm, while the mean increase in endometrial thickness in G-CSF treated group was 3.27 mm.

Conclusion

This study has evidence of better results in improving endometrial thickness in infertile women by using vaginal sildenafil with respect to endometrial growth with an intrauterine infusion of granulocyte colony-stimulating factor (filgrastim, G-CSF).

## Introduction

Infertility presently is an emanating preventive reproductive health issue with vast psychopathological and socio-emotional repercussions affecting almost every community. They are affected in terms of their respect, status, authority, loss of will to live owing to a sense of failure, and exclusion from the daily spheres of life [1,2].

Epidemiologically, In India, primary infertility ranges between 4-17% [3]. Numbers from census data of India (1999-01 and 2009-11) show that fertility, at the pan country level, had a decline of 17.7 % in the general fertility rate (GFR), and it varied from 17.4% and 15.9% in rural and urban areas, respectively. More recently, the pattern continued, and the average GFR had a decline of 20.6% during the successive ten years [4].

As for a favorable implantation outcome, an appositely assembled endometrial lining should be present during the menstrual cycle, making it the most critical factor for predicting a patient's ability to achieve a healthy pregnancy. This receptivity is indirectly related to and determined by the blood flow to the endometrial tissue. Moreover, apposite proliferative and secretory changes of the endometrium (like stromal cell enlargement with active mitosis, proliferation and coiling of the uterine glands, elongation of the spiral arteries, and activation of decidual immune cells [T lymphocytes and natural killer cells]) ensures successful implantation of a healthy blastocyst [5,6]. To reach the endometrium, these factors require a sufficient uterine blood supply. Most of the case studies assent practically that the functional layer has to reach an acceptable thickness of more than 7 mm for successful pregnancy [6,7]. Some of the suggested treatments for the thin endometrium are by use of intrauterine instillation with granulocyte-colony stimulating factor (G-CSF) [8] or vaginal sildenafil [9].

With advancements in molecular biochemistry, the isolation of independent growth factors has become more accessible. A purified *Escherichia coli* expressed recombinant factor called recombinant human granulocyte-colony stimulating factor (rhG-CSF) is frequently indicated in managing some hematological indications [10-12]. It has now been clinically proven to improve the chances of conception in patients who have failed in vitro fertilization (IVF) owing to endometrial growth or endocrinal function insufficiency [12-16], and it is even being recognized as a key component in preventing repeated miscarriages without affecting embryo genetics.

Rationalizing this, Gleicher et al. proposed a methodology of stimulating endometrial growth by intrauterine injection (IUI) of G-CSF (or filgrastim) [14]. G-CSF is a cytokine that only affects the hematopoietic system, which is generated by a large group of body cells (those are bone marrow, stromal cells, fibroblasts, etc.) Which upstroke the differentiation as well as neutrophil growth in the bone marrow and regulation of their release into the circulation. G-CSF enhances phagocytosis in the macrophages of decidual cells in adult neutrophils [8], which finally affects the implantation [11]. Immunological activities of G-CSF include dendritic cell recruitment, enhancement of type 2 T-helper cell (Th-2) cytokine production, activation of T regulatory cells, and different proangiogenic effects up-activation [12].

By inhibition of the enzyme phosphodiesterase type 5 enzyme (PDE-5), which is responsible for the degradation of guanosine 3',5'-cyclic monophosphate (cGMP), sildenafil boosts the impact of nitric oxide. Sildenafil is a selective inhibitor of the type V cGMP- specific phosphodiesterase. Sildenafil keeps cGMP levels high and promotes endometrial thickness by providing a suitable environment (i.e., by causing vascular relaxation and enhanced blood flow directed towards it) [17,18].

## Materials and methods

Study design 

It was a randomized controlled trial (RCT) study that was single-centered, open-labeled, and conducted over a two months study period between September 2021 to November 2021.

Ethical clearance

The study was approved by the Medical Ethics Committee of Datta Meghe Institute of Medical Sciences Deemed to be University (ethical approval code: DMIMS(DU)/IEC/2021/543). The objective of the study was presented to eligible participants, and consent was acquired in the local language or their preferred language (if they failed to comprehend the procedure). A formal informed consent form was signed by all study participants.

Study setting

Premenopausal women seeking infertility treatment were recruited from the gynecology outpatient department (OPD) of the hospital. Scrutinizing heads also kept a check on any possible medical records to boost recruiting.

Study sample size

Based on studies by Krejcie and Morgan [19], the sample size was calculated from the total number of patients in any consecutive six months in OPD. As calculated statistically, an expected total of 70 patients with infertility (primary and secondary) had to be recruited into two groups of vaginal sildenafil takers and intrauterine G-CSF, including 35 patients each over the study period of two months.

Potential participants were identified through a review of all patients attending infertility treatment sessions during the study period, as well as information from the doctors in charge of each OPD unit. Patients were provided patient information leaflets (PILs) prior to meeting with the researcher in person.

Individuals were approached for discussion and possible enrollment in the research at their regular gynecological or artificial reproductive technology (ART) unit appointment. Potential participants had the freedom to reflect on the material presented both verbally and in the PIL.

Since the length and timing of this preceding interview were determined by the patient's awareness of their underlying condition and the study endeavor, there was no set time limit. Each patient included in the research provided written permission (in English or the local language).

Baseline assessment 

All patients received a thorough medical history and physical examination, as well as a baseline laboratory evaluation, a vaginal ultrasound, and hysterosalpingography (HSG).

Randomization

Patients who fulfilled the inclusion criteria were randomly assigned to one of two intensive outpatient programs using simple randomization of inpatient department (IPD) number by SPSS software, IBM (Armonk, USA). The research began on the patient's sixth day of the menstrual cycle or the next day after the last menses of the current cycle.

Interventions

Group A included 35 female patients who were asked to self-administer non-clinically vaginal sildenafil citrate 25 mg every six hours from day seven to day 12 of the menstrual cycle. The patients were asked to report to the clinic on the last day of menses (i.e., day six of the menstrual cycle) and the day after the last drug dose (i.e., day 12 of the cycle) for the baseline endometrial thickness measurement and the changed endometrial thickness measurement. The patient was called last time, two days after (i.e., day 14 of the cycle), for the final reading of the ET.

Group B included 35 female patients who had their baseline ET measurement on the last day of menses (i.e., day six of the menstrual cycle) and received intrauterine G-CSF injection according to Gleicher et al.'s procedure [14] with full bladder before transfer (filgrastim 300 mcg or any other bioequivalent of 300 µg [300 mcg/1 mL] of G-CSF) via IUI catheter four days after the last day of menses (i.e., day 10 of the menstrual cycle) and ET was measured on two days with a gap of a couple of days (i.e., on day 12 and day 14 of the menstrual cycle).

Both the groups' participants received a modified Standard Protocol Items: Recommendations for Interventional Trials (SPIRIT) diagram leaflet stuck to their usual follow-up prescription/follow-up booklet, which had all the chronological appointment days mentioned with a self-checklist box. 

The patients were followed up on reporting to the clinic on respective side effects and any other inconvenience caused. 

Procedure for measuring endometrial thickness 

A complete infertility workup was done for all patients. Data was collected using a preformed pre-tested form. Endometrial thickness was measured from one echogenic border to another echogenic border across the endometrial cavity on a midline image of the sagittal plane, and the greatest distance between the myometrial and endometrial interfaces was determined using the uterus' central longitudinal axis and calipers placed on the endometrium's outer wall. All patients had a transvaginal scan (non-invasive type) using GE Logiq P5 Machine (Boston, USA). A 5 MHz ultrasound probe was used to assess the thickness of the endometrium. 

Statistical analysis

Descriptive statistics were applied to summarize the demographic data. Results are reported as means with standard deviations. Shapiro-Wilk test was used to find the evidence of non-normality of data collected in the study variables. Paired and unpaired t-tests were applied to detect significant differences among stratifications. Software SPSS v23 was used to analyze the statistical data. P-value <0.05 was considered significant.

## Results

**Figure 1:**
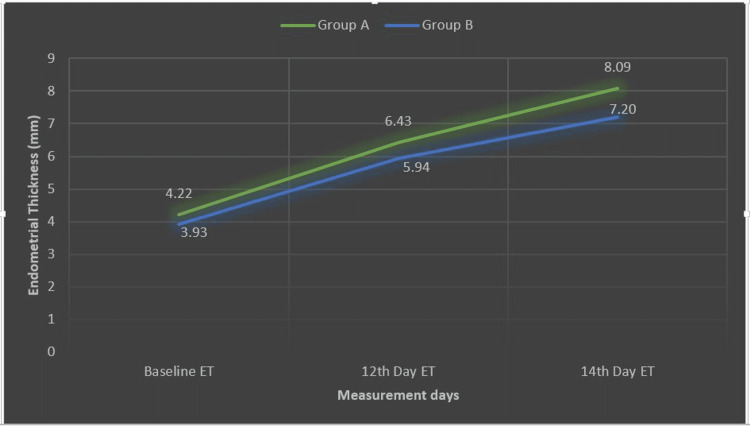
Mean increment in endometrial thickness in study groups on 12th and 14th days Group A: study group (green) treated with vaginal sildenafil Group B: study group (blue) treated with granulocyte-colony stimulating factor (G-CSF) ET - endometrial thickness

According to Tables 1-3, the paired-samples t-test showed a significant difference in the measurement of endometrial thickness for baseline (m=4.226 mm, SD=0.760) and 14^th^ day of endometrial thickness after the intervention of vaginal sildenafil in group A (m=8.094, SD=1.0849) conditions; t(340)=-19.46, p=0.000 (CI: -4.27 to -3.46). Whereas endometrial thickness for filgrastim intervention group B was less at baseline (m=3.934 mm, SD=0.655) than on the 14^th^ day (m=7.206 mm, SD=0.916). This improvement, 3.27 mm, 95% CI (-3.69 to -2.85) was also statistically significant, t(34)=-15.86, p=0.000. These results are shown graphically in Figure 1; mean endometrial thickness after intervention at the 14^th^ day was significantly higher in group A (8.09 mm), which was exposed to vaginal sildenafil treatment, than in group B mean endometrial thickness after intervention at the 14^th^ day (7.2 mm), which was exposed to filgrastim treatment.

**Table 1:** Mean baseline characteristics of interventional groups BMI - body mass index measured as weight in kilograms divided by height in squared meters.

Measurement points	Group A (mean ± SD)	Group B (mean ± SD)
Age (years) at screening	31.69 ± 6.87	31.34 ± 6.37
BMI	23.32 ± 2.38	22.66 ± 2.60

**Table 2:** Mean endometrial thickness on different days for the interventional groups ET - endometrial thickness

Measurement points	Group A (mean ± SD)	Group B (mean ± SD)
Baseline ET, mm	4.22 ± 0.76	3.93 ± 0.65
12^th^ day ET, mm	6.43 ± 0.98	5.94 ± 0.82
14^th^ day ET, mm	8.09 ± 1.08	7.20 ± 0.91

**Table 3:** Mean difference of endometrial thickness post-treatment in the interventional groups * significant ET - endometrial thickness

Study parameter	Group A		Group B	
	Mean difference	p-value	Mean difference	p-value
Baseline ET - 14^th^ day ET	-3.86	0.00*	-3.27	0.00*

For testing the hypothesis that the difference in endometrial thickness or increment at the end of the 14^th^ day of intervention of sildenafil in group A (n=35, m=3.869, SD=1.1762) was unequal and more than that of endometrial thickness increment at the end of 14^th^ day of intervention with filgrastim in group B (n=35, m=3.271, SD=1.2206), an unpaired samples t-test was performed. Prior to conducting the analysis, the assumption of normally distributed difference scores was examined by Shapiro-Wilk's test. The assumption was considered satisfied (W=0.988, p=0.724), suggesting that the independent samples t-test can be applied in the case. The test for assumption equality of variances was checked by Levene's test for equality. Levene's test indicated equal variances (F=0.003, p=0.953). The null hypothesis that the use of vaginal sildenafil citrate exerts an effect similar to that of the G-CSF intrauterine injection in increasing endometrial thickness was rejected, t(68)=2.08, p<0.05. Thus, the group A participants' increase in endometrial thickness was statistically significantly higher than that of group B participants' increase of endometrial thickness. Cohen's d was estimated at 0.5 which is a medium effect based on Cohen's (1992) guidelines.

## Discussion

As described in Table 1, the mean age of patients was 31.51±6.6 years (25-38 years) with a healthy BMI (m=22.9, SD=2.49). This finding was comparable to the study of Mishra et al. [20]. The findings of the present study are also in accordance with Aleyasin et al., who conducted a prospective study on 28 women to find improvement in endometrial thickness in 71.4% of women in the age group from 20 to 40 years [21]. A study by Adamson et al. found the mean age was 25.9 years which was similar to the present study [22]. The present study was carried out on a younger population in contrast to studies by Gliescher et al. [13] and Kunicki et al. [16]. The patients included in the study had a normal average baseline endometrial thickness of 4.1 mm on the sixth day of their respective cycles, signifying that their endometrial thickness was of normal measurement and devoid of any physiological as well as pathological factors that may interfere with the intervention given to them.

In the study, it was observed that percentage of women in group A on day nine who had 3-4 mm ET was 34.3%, 5.6-6 mm ET on day 12 was 20%, and 8.6-9.0 mm ET on day 14 was 25.7%. All these women were prescribed sildenafil as they had thin endometrium.

Out of the study group (n=40), unfortunately, one woman was lost in follow-up, and four women opted out of the intervention mainly due to non-compliant behavior. This group had the most number of opt-out participants. So a total of five women from the pre-intervention group could not be included in the post-intervention group.

Also, in group B, 38 women were included, but unfortunately, three women were lost in follow-up. So a total of three women from the pre-intervention group could not be included in the post-intervention group. The percentage of women in group B on day nine who had 3-4 mm ET was 31.4%, 5.6-6 mm ET on day 12 was 28.6%, and 7.6-8.0 mm ET on day 14 was 25.7%. All these women were prescribed filgrastim injection as they had thin endometrium.

There is a mean increase of 2.21 mm and 3.87 mm on day 12 and 14, respectively, in group A (p<0.05), and a mean increase of 2.01 mm and 3.27 mm on day 12 and 14, respectively, in group B (p<0.05). Therefore it can be concluded that both sildenafil, as well as filgrastim, causes a statistically significant increase in endometrial thickness. Observations made in this study were supported by the studies listed in Table 4.

**Table 4:** The comparison of results of different studies regarding pre- and post-infusion endometrial thickness ET - endometrial thickness; G-CSF - granulocyte colony-stimulating factor *significant as per the researcher's setting

Study	Drug intervention	Baseline ET (mm)	Post-infusion ET (mm)	Difference (mm)	p-value
This study	vaginal sildenafil	4.23 ± 0.76	8.09 ± 1.08	3.87 ± 1.32	p < 0.05
Al-Assadi et al.[23]	vaginal sildenafil	5.21 ± 1.28	7.64 ± 2.02	2.43 ± 2.39	p < 0.001
Takasaki et al. [24]	vaginal sildenafil	-			p < 0.001
Razieh Dehghani et al. [25]	vaginal sildenafil	Mean post-infusion ET = 9.8mm			p < 0.0001
Mangal et al.[26]	vaginal sildenafil	5.42	9.42	4.0	p < 0.14*
This study	G-CSF	3.93 ± 0.65	7.20 ± 0.91	3.27 ± 1.12	p < 0.05
Gleicher et al. [13]	G-CSF	5.2 ± 1.4	9.3 ± 2.1	2.9 ± 2.0	p < 0.001
Michal et al. [16]	G-CSF	6.74 ± 1.75	8.42 ± 1.73	1.68 ± 1.05	p < 0.0001
Vineet et al. [20]	G-CSF	5.86 ± 0.58	6.58 ± 0.84	0.72 ± 0.26	p < 0.001
Davari-tanha et al. [27]	G-CSF	3.6 ± 0.98	7.12 ± 0.84	3.53 ± 0.88	p < 0.001

The significant increase in endometrial thickness by administration of vaginal sildenafil with respect to the administration of granulocyte colony-stimulating factor in the study population is assignable to its mechanism of action [17,18], less side effects [9], and as seen from the study, self-administrative dosage owing to less dependency on any health professional and its cost-effectiveness.

Limitations

The sample size was relatively small for this study. Moreover, attrition bias may be present due to the loss of follow-up of patients.

## Conclusions

The following study was focused on finding the better alternative between two (G-CSF treatment and vaginal sildenafil) of the many treatment choices for improving the pregnancy (both chemical and clinical) of an infertile couple by increasing the endometrial thickness (ET). In various research projects, there had been a significant correlation between these individual drugs and positive pregnancy rates. Hence, this study took the step forward by comparing these drugs in parallel with the baseline profiles. This randomized controlled trial examined the associations of these medicines using proven measuring techniques and tested the usefulness of these drugs after the statistical threshold was reached. The mean increase in endometrial thickness in the per vaginal sildenafil treated group was 3.87 mm, while the mean increase in endometrial thickness in G-CSF treated group was 3.27 mm. Therefore, vaginal administration of sildenafil may be more effective in increasing endometrial thickness than the intrauterine infusion of G-CSF.
